# EHMTI-0377. The influence of genetic constitution on migraine drug responses

**DOI:** 10.1186/1129-2377-15-S1-M3

**Published:** 2014-09-18

**Authors:** AF Christensen, AL Esserlind, T Werge, H Stefansson, J Olesen

**Affiliations:** 1 Dep. of Neurology Glostrup University Hospital University of Copenhagen, Danish Headache Center, Copenhagen, Denmark; 2 Mental Health Center Sct. Hans University of Copenhagen, Institute of Biological Psychiatry, Roskilde, Denmark; 3 Sturlugata 8, deCODE Genetics, Reykjavik, Iceland

## Background

Specific acute treatments of migraine are 5HT1B/D receptor agonists, i.e. triptans and ergotamine. No migraine prophylactic drugs are specific to migraine. Prophylactic drugs are selected by time consuming “trial and error”. Personalized treatment is therefore much needed.

## Aim

The objective of this study was to test the effect of 12 common SNPs significantly associated with migraine on migraine drug responses.

## Methods

Semi-structured migraine interviews, blood samples and genotyping were performed on 1806 unrelated migraine cases recruited from the Danish Headache Center. Association analyses were carried out using logistic regression and ORs were calculated assuming an additive model for risk. The effect on drug responses was tested for a combined genetic score and for each of the 12 SNPs.

## Results

A higher combined genetic score, and a single SNP, rs2651899 in PRDM16, were significantly associated with efficacy of triptans with an OR of success of 2.6 and 1.3, respectively. A number of SNPs showed nominal preferential association with the efficacy of triptans and others with prophylactic drugs.

## Conclusion

We show for the first time an association between genetic constitution and migraine drug response. This is a first step towards future individualized medicine.

No conflict of interest.

